# Remedial Aspect of Zinc Oxide Nanoparticles Against *Serratia Marcescens* and *Enterococcus Faecalis*

**DOI:** 10.3389/fphar.2022.891304

**Published:** 2022-06-07

**Authors:** Sinouvassane Djearamane, Zhe Chi Loh, Jun Jie Lee, Ling Shing Wong, Ranjithkumar Rajamani, Priscy Alfredo Luque, Piyush Kumar Gupta, Sharolynne Xiao Tong Liang

**Affiliations:** ^1^ Department of Biomedical Science, Universiti Tunku Abdul Rahman (UTAR), Kampar, Malaysia; ^2^ Faculty of Health and Life Sciences, INTI International University, Nilai, Malaysia; ^3^ Viyen Biotech LLP, Coimbatore, India; ^4^ Faculty of Engineering, Architecture, and Design, Autonomous University of Baja California, Mexicali, Mexico; ^5^ Department of Life Sciences, School of Basic Sciences and Research, Sharda University, Greater Noida, India; ^6^ Department of Biotechnology, Graphic Era Deemed to be University, Dehradun, India

**Keywords:** zinc oxide nanoparticles, antibacterial agent, *S. marcescens*, *E. faecalis*, growth inhibition

## Abstract

Zinc oxide nanoparticles (ZnO NPs) have been widely used in biomedical applications due to their high biocompatibility and low toxicity to humans. The present work aimed to investigate the antibacterial effects of different concentrations of ZnO NPs on two opportunistic pathogens, *Serratia marcescens* and *Enterococcus faecalis*. The surface interaction between nanoparticles and bacterial cell wall, and the subsequent morphological alterations on the bacterial surface, were examined through Fourier transform infrared spectroscopy and scanning electron microscope. The energy dispersive X-ray analysis was used to confirm the elemental composition of ZnO NPs and the cellular accumulation of ZnO NPs in bacteria. The growth-inhibitory test demonstrated a dose-dependent growth inhibitory effect of ZnO NPs against both the test bacteria, as the higher concentration of nanoparticles caused the higher bacterial growth inhibition. The results showed that ZnO NPs caused a higher growth inhibition (63.50 ± 2.50%) on the Gram-positive bacterium *E*. *faecalis* compared to the Gram-negative bacterium *S. marcescens* (51.27 ± 4.56%). Fourier transform infrared spectrum revealed the possible involvement of hydroxyl, carboxyl, amides, methylene, and phosphate groups from the biomolecules of bacterial cell wall such as proteins, carbohydrates, lipids, and phospholipids in the interaction of ZnO NPs on bacterial cell surface. Energy dispersive X-ray analysis showed the higher accumulation of ZnO NPs in *E. faecalis* than *S. marcescens* analogous to the bacterial growth inhibition. Scanning electron microscopy images confirmed the antibacterial properties of ZnO NPs, showing the loss of integrity of cell membrane and distortion of bacterial cells. Hence, the potential of ZnO NP as an antibacterial agent against *S. marcescens* and *E. faecalis* has been confirmed.

## Introduction

New antimicrobial agents against pathogenic bacterial strains have become extremely challenging due to the antibiotic resistance and mutations that lead to new strains of the pathogen (Fair and Tor, 2014; Sirelkhatim et al., 2015; Ventola, 2015; Aslam et al., 2018; Fisher et al., 2020). The currently available antibiotics are designed to fight against bacteria through the inhibition of DNA replication, cell wall synthesis, and translational machinery; nevertheless, bacterial resistance might be developed against each of these antibiotics (Magiorakos et al., 2012; Li et al., 2013: Nurit et al., 2015). The field of nanotechnology, which provides nanoscale materials, can be an effective solution in controlling the bacterial infections. The unique chemical and physical attributes of nanoparticles (NPs) play a vital role in therapeutic applications in the biomedical field (Patra et al., 2018; Elena et al., 2020; Maheswaran et al., 2021). Generally, NPs have a larger surface area to volume ratio than bulk materials. Subsequently, the nanoscale size of materials allows more atoms to be exposed on the bacteria cell surface and thus can easily exert their bactericidal mechanisms to the target cell (Seil and Webster, 2012; Dadi et al., 2019; Dikshit et al., 2021).

Various metal nanoparticles such as zinc, silver, gold, titanium, manganese, platinum, copper, iron, and magnesium NPs have been reported as promising agents against the pathogenic microorganisms over commercial antibiotics (Wang et al., 2017; Djearamane et al., 2019; Sánchez-López et al., 2020; Yaqoob et al., 2020). Among the metal nanoparticles, zinc oxide nanoparticles (ZnO NPs) have been invstigated for multiple biomedical applications due to their lower toxicity to humans (Fernando et al., 2018; Vijayakumar et al., 2018). ZnO NPs are reported to have antibacterial activity on various human and animal pathogenic bacteria (Alekish et al., 2018). The higher surface to volume ratio of ZnO NP is believed to be responsible for its greater bacteriostatic effects (Siddiqi et al., 2018). The treatment of bacteria with ZnO NPs causes induction of oxidative stress through increased production of reactive oxygen species and the subsequent peroxidation of lipid bilayer in cell membrane, disturbing the integrity of the bacterial cell wall (Kadiyala et al., 2018; Tiwari et al., 2018:; Djearamane et al., 2020). The attachment of ZnO NPs on the bacterial surface is also reported to cause damage to the cell membrane and leakage of cytoplasmic components, resulting in bacterial cell death (Alekish et al., 2018; Liang et al., 2020; Dhanasekaran et al., 2022).

In this study, two multidrug resistant bacteria which commonly cause opportunistic infections, namely *Serratia marcescens* and *Enterococcus faecalis*, are used as the test organisms to explore the potential of ZnO NPs to be applied as an antibacterial agent against the infections caused by them.

*S. marcescens* is a rod-shaped Gram-negative bacterium which is commonly associated with opportunistic infections, especially among immunocompromised patients, ranging from abscess formation, granulomatous lesions, to eye, respiratory, and urinary tract infections. If the infection is not diagnosed and treated early, it can lead to serious complications such as sepsis and meningitis (Kim et al., 2015; Ray et al., 2015; Abreo and Altier 2019). It is a Gram-negative bacteria that causes invasive burn wound infections and accounts for 11% of burn-related surgical wound infections (Khayyat et al., 2021). The ability of *S. marcescens* to produce β-lactamase facilitated the resistance towards broad-spectrum antibiotics including β-lactams, aminoglycosides, and fluoroquinolones, making the treatment and management of the infections caused by *S. marcescens* very complicated (Dhusia et al., 2019; Khayyat et al., 2021).

The Gram-positive bacterium *E. faecalis* usually does not cause infection in healthy individuals. However, people with immunocompromised or underlying health conditions are more prone to *E. faecalis* infections including wound infections, urinary tract infections, bacteremia, endocarditis, and meningitis (Ayobami et al., 2020). *Enterococcus* species are reported to have a wide range of resistant genes which make them resistance to first-line antibiotics (Shiadeh et al., 2019). Recently, there has been an increase in the number of *E. faecalis* strains becoming multi-drug resistant (Kau et al., 2005; Castilho et al., 2013).

This study is aimed to determine the antibacterial effect of ZnO NPs against *S. marcescens* and *E. faecalis* by investigating the bacterial growth inhibition and the interaction between NPs and bacteria.

## Materials and Methods

### Chemicals and Nanoparticles

Zinc oxide nanoparticles with size <100 nm, phosphate buffer saline (PBS), 10 X, and glutaraldehyde were obtained from Sigma-Aldrich, United States of America. The antibiotics chloramphenicol and ampicillin were purchased from Bio Basic Inc., Canada. The bacterial culture medium Luria Bertani (LB) was purchased from Laboratories CONDA, Spain.

### Characterization of ZnO NPs

Scanning electron microscope with energy dispersive X-ray (SEM- EDX) (JOEL JSM 6710F, Japan) was used to characterize the surface morphology and size of ZnO NPs through SEM operated at an acceleration voltage of 4 kV with a working distance of 4.7 nm, while the elemental composition of ZnO NPs was studied by EDX analysis. X-ray diffractometer (XRD) (Lab X, SHIMADZU, XRD-6000, Japan), operated at an angle of 2θ with 40 V and 30 mA current, was applied to confirm the crystalline structure and size of the nanomaterial. Further, Fourier transform infrared (FTIR) spectroscopy (Perkin-Elmer Spectrum RX1, United States) was used to confirm the chemical composition of ZnO NPs.

### Preparation of ZnO NPs Suspension

A stock solution of 320 μg/ml ZnO NPs was prepared by suspending ZnO nanopowder in LB broth, mixed homogenously by vortex, and diluted with LB broth to prepare the working concentrations of ZnO NPs.

### Bacterial Culture

The Gram-negative bacterium *S. marcescens* (ATCC 43862) and the Gram-positive bacterium *E. faecalis* (ATCC 29121) were obtained from the Faculty of Science, Universiti Tunku Abdul Rahman and then subcultured to the mid-log phase in LB broth for further utilization.

### Exposure of Bacteria to ZnO NPs

A 5 ml of mid-log phase suspension of *S. marcescens* with an optical density of 0.05 at 600 nm (OD600) was exposed to 5 ml of ZnO NPs to obtain the final concentrations of 5, 10, 20, 40, 80, and 160 μg/ml of ZnO NPs in 15 ml centrifuge tubes and incubated for 24 h at 37°C. Simlarly, the mid-log phase suspension of *E. faecalis* with the same OD600 of 0.05 was treated with various concentrations of ZnO NPs ranging from 5 to 160 μg/ml of ZnO NPs for 24 h, but incubated at 35°C. The bacterial suspension without ZnO NPs was used as the negative control. The suspension of *S. marcescens* treated with 0.8 mg/ml of chloramphenicol and *E. faecalis* exposed to 1.0 μg/ml of ampicillin was used as the positive control, respectively (Conceição et al., 2014; Ray et al., 2017).

### Growth Inhibition Test

Turbidity test was conducted to investigate the antibacterial effect of ZnO NPs on *S. marcescens* and *E. faecalis* by determining the optical density of bacterial suspensions treated with 5, 10, 20, 40, 80, and 160 μg/ml of ZnO NPs by spectrophotometer at 600 nm (Libra S4, Biochrom, UK) along with negative and positive controls. LB broth was used as the blank. The absorbance from the respective concentration of ZnO NPs was subtracted from the test readings to avoid the interference by the NPs. Then, the absorbance values were compared to calculate the percentage of bacterial growth inhibition using equation 1 (Eq. 1).

Percentage of growth inhibition = (OD600 negative control–OD600 test)/OD600 negative control × 100 (Eq.1).

### Investigation of Surface Interaction of ZnO NPs on Bacterial Cell Wall

The FTIR spectroscopy was used to investigate the involvement of functional groups from the bacterial cell wall for the interaction of ZnO NPs on the bacterial surface. A volume of 25 ml of the bacterial suspensions treated with 160 μg/ml of ZnO NPs for 24 h, along with the negative control, were centrifuged for 10 min at 6,000 *g* to obtain the pellets. The pellets were then washed with 1X PBS three times to remove the unbound NPs, freeze-dried, and subjected to FTIR spectrum analysis at 4,000 to 400 cm^−1^.

### Scanning Electron Microscopy With Energy Dispersive X-Ray Analysis

The SEM images were obtained to investigate the morphological damage caused by ZnO NPs on treated bacteria, while EDX was performed to identify the accumulation of ZnO NPs in bacterial cells. A volume of 25 ml of the bacterial suspensions treated with 160 μg/ml of ZnO NPs for 24 h, along with the negative control, were centrifuged for 10 min at 6,000 g to obtain the pellets. The pellets were then washed with 1X PBS three times and treated with 2.5% of glutaraldehyde in 1X PBS for overnight fixing. The next day, the samples were centrifuged and washed three times with 1X PBS for 10 min at 6,000 *g*. This washing process was repeated using distilled water. Then, the samples were dehydrated by a series of different concentrations of ethanol (25%, 50%, 75%, 95%, and 100%). The dehydration process with 100% ethanol was repeated three times, followed by critical point drying and sputter coating. The samples were then analyzed under SEM-EDX (JOEL JSM 6710F, Japan).

### Statistical Analysis

Statistical analysis was conducted to identify the variances when different concentrations of ZnO NPs interacted with *S. marcescens* and *E. faecalis.* The tests were done in triplicates (n = 3), and the data are presented as mean ± standard deviation. All data were analyzed by one-way analysis of variance (ANOVA) and the statistical significance was reported at *p* < 0.05.

## Results and Discussion

### Characterization of ZnO NPs

**FIGURE 1:**
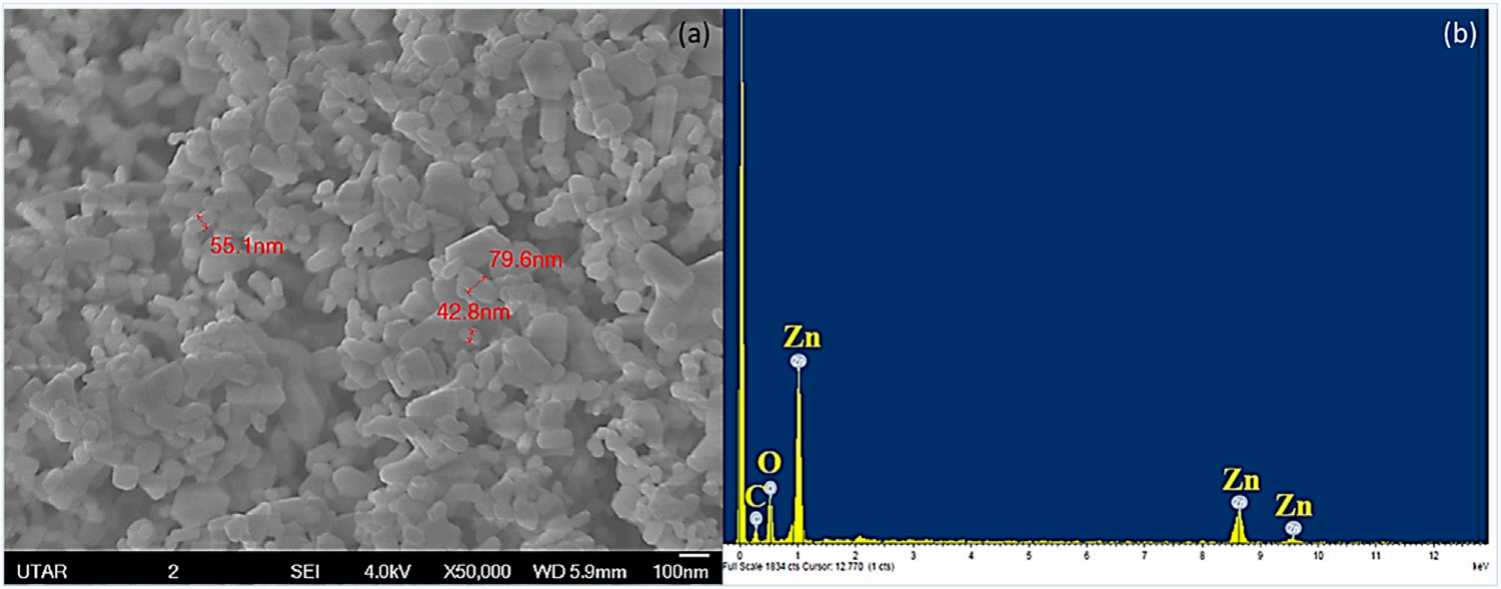
SEM-EDX of ZnO NPs. SEM analysis showing the spherical and rod-shaped NPs **(a)** and EDX analysis showing the presence of zinc and oxygen in ZnO NPs **(b)**.

The surface morphology and elemental composition of ZnO NPs were analysed by SEM-EDX. Figure 1(a) shows that the ZnO NPs were spherical particles with a mixture of rods, while the particle size ranged from 42.8 to 79.6 nm with a mean size of 59.1 nm. The EDX spectrum depicted in Figure 1(b) confirmed the presence of ZnO NPs by the peaks of zinc and oxygen molecules. The presence of zinc, oxygen, and carbon elements from EDX analysis indicated that ZnO NPs used in the present study were free from impurities. The carbon that appeared in the EDX spectrum may be from the carbon tape used for sample preparation (Varadavenkatesan et al., 2019).

**FIGURE 2:**
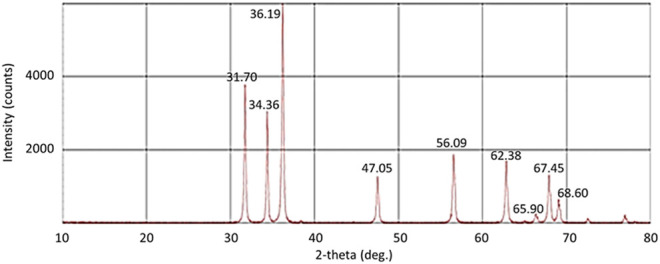
XRD spectrum of ZnO NPs.

**FIGURE 3:**
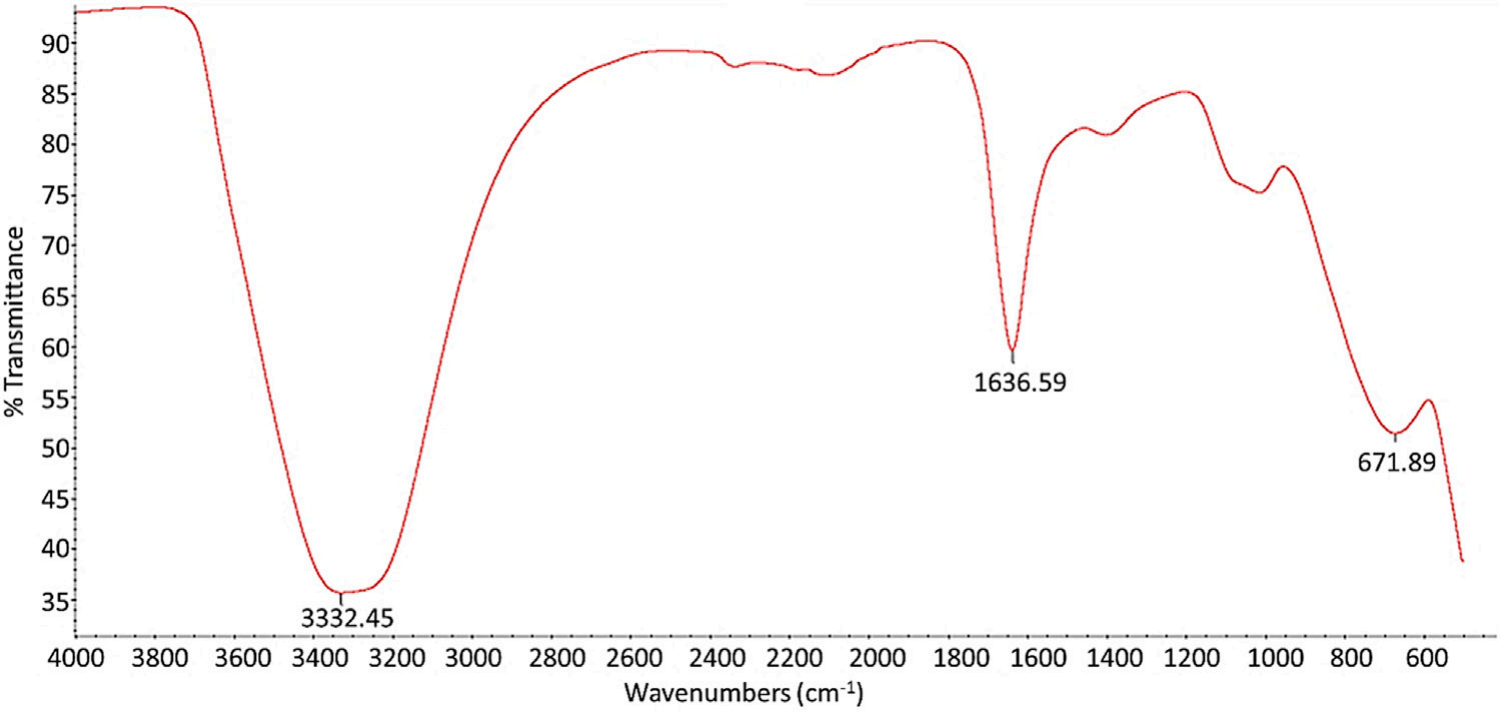
FTIR spectrum of ZnO NPs.

The strongest diffraction peaks at 31.7°, 34.36°, and 36.19° and also the peaks at 47.05°, 56.09°, 62.38°, 65.90°, 67.45°, and 68.60° displayed in the XRD spectrum of ZnO NPs (Figure 2) corresponded to the characteristic hexagonal wurtzite crystalline structure of ZnO NPs, according to the JCPDS database card number [01-070-2551] (Ramesh et al., 2015; Kumar and Gautam, 2019; Muhammad et al., 2019). The XRD spectrum of ZnO NPs was also utilized to calculate the crystalline size of ZnO NPs using Scherrer’s equation. The crystalline size was found to be at the range of 40–47 nm with a mean size of 43 nm. In addition, FITR spectrum of ZnO NPs was used to confirm the composition of ZnO NPs. As shown in Figure 3, FTIR spectrum of ZnO NPs obtained in this study illustrated the peak absorbance at 3332.45, 1636.59, and 671.88 cm^−1^. Earlier studies have reported Zn-O stretching at 1634.00 cm^−1^ (Kumar and Rani, 2013), stretching of ZnO NPs at the range of 400–800 cm^−1^ (Ramesh et al., 2015), and stretching of H-O-H vibrations between 3400 cm^−1^ and 3600 cm^−1^ (Sukri et al., 2019).

### Growth Inhibition

**FIGURE 4:**
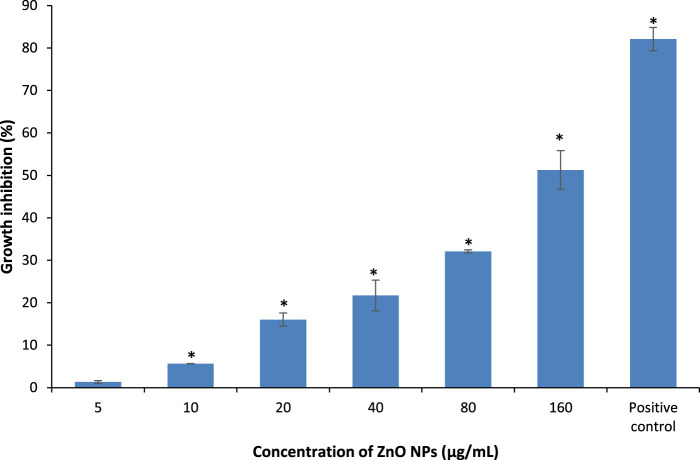
Percentage of growth inhibition of *S. marcescens* after treatment with different concentrations of ZnO NPs for 24 h at 37°C in LB broth. * denotes the significant difference between the negative control and the bacterial suspension treated with different concentrations of ZnO NPs at *p* < 0.05.

**FIGURE 5:**
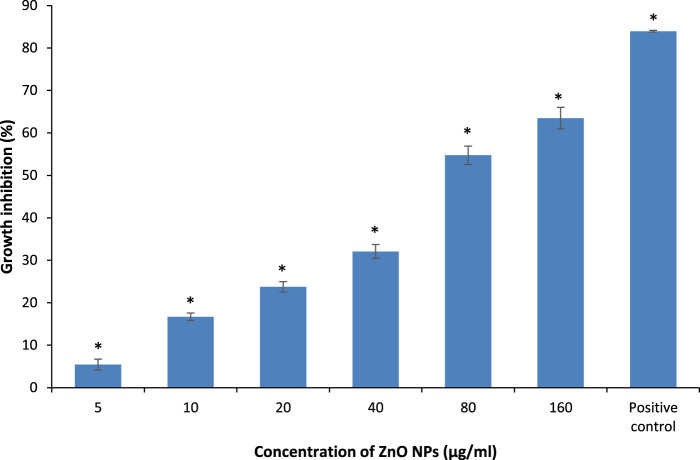
Percentage of growth inhibition on *E. faecalis* upon treatment with different concentrations of ZnO NPs for 24 h at 35°C in LB broth. * indicates the significant difference between the negative control and the bacterial suspension treatment with different concentrations of ZnO NPs at *p* < 0.05.

The absorbance of the bacterial suspensions were measured at 600 nm upon treatment with different concentrations of ZnO NPs from 5 to 160 μg/ml for 24 h, along with the negative and positive controls for the respective bacteria. The treatment of ZnO NPs resulted in a dose-dependent bacterial growth inhibition, as the higher the concentrations of ZnO NPs, the greater the percentage of growth inhibition on *S. marcescens* (Figure 4) and *E. faecalis* (Figure 5).

The percentage of growth inhibition at 5, 10, 20, 40, 80, and 160 μg/ml of ZnO NPs against *S. marcescens* were 1.34 ± 0.32, 5.65 ± 0.04, 16.03 ± 1.54, 21.73 ± 3.6, 32.1 ± 0.34, and 51.27 ± 4.56% respectively. Similarly, ZnO NPs caused 5.42 ± 1.27, 16.69 ± 0.88, 23.74 ± 1.23, 32.07 ± 1.65, 54.73 ± 2.15, and 63.50 ± 2.50% of growth inhibition at 5, 10, 20, 40, 80, and 160 μg/ml of ZnO NPs respectively on *E. faecalis*. The percentage of growth inhibition in positive control was 82.78 ± 1.08% and 83.93 ± 0.18% for *S. marcescens* (with 0.8 mg/ml of chloramphenicol) and *E. faecalis* (with 1.0 μg/ml of ampicillin), respectively.

The results demonstrated a significant (*p* < 0.05) bacterial growth inhibition from 10 to 160 μg/ml of ZnO NPs, however, no significant growth inhibition was detected at 5 μg/ml of ZnO NPs for *S. marcescens*. Whereas, the treatment of ZnO NPs caused significant (*p* < 0.05) growth inhibition on *E*. *faecalis* for all the tested concentrations from 5 to 160 μg/ml.

Our results indicate a better antibacterial activity of ZnO NPs on Gram-positive bacterium compared with Gram-negative bacterium as the highest concentration of ZnO NPs (160 μg/ml) caused 63.50 ± 2.50% of growth inhibition on *E*. *faecalis* compared to 51.27 ± 4.56% on *S. marcescens* as the maximum growth inhibition. Supporting the present results, Azam et al. (2012) reported a higher tolerance of Gram-negative bacteria over Gram-positive bacteria to ZnO NPs as the treatment of ZnO NPs caused 16 and 24% lower inhibition zone sizes on Gram-negative bacterial strains of *P. aeruginosa* and *E. coli* compared to Gram-positive bacterial strains of *S. aureus* and *B. subtilis*. Further, Premanathan et al. (2012) evidenced a more profound effect of ZnO NPs on Gram-positive bacterial strains compared to Gram-negative bacterial strains. Earlier studies by Chandra et al. (2019) and Gunalan et al. (2012) reported the growth inhibition of *S. marcescens* by ZnO NPs with the minimum inhibitory concentration of 64 μg/ml and 1.6 mM, respectively, at 24 h through disc diffusion assay.

Similar to our present results, previous studies have demonstrated a dose-dependent growth inhibition on *E. faecalis* using agar diffusion by gelatin-coated ZnO NPs and ZnO NPs, respectively (Dowlatababdi et al., 2017; Divya et al., 2018; Sukri et al. (2019) confirmed a higher antibacterial potential of green synthesized ZnO NPs on *E. faecalis* by reporting 22.09 μg/ml of ZnO NPs as the minimum inhibitory concentration inhibiting 50% growth for *E. faecalis* compared to 64.53 μg/ml for Gram-negative bacterium *E. coli*. A study by Nilavukkarasi et al. (2020) demonstrated a more effective antibacterial action of ZnO NPs on Gram-positive bacterium *S. epidermidis* than *S. paratyphi*, which is a Gram-negative bacterium.

Simlar to the present results, our earlier studies also demonstrated a dose-dependent bacterial growth inhibition by ZnO NPs with a higher growth inhibition of 81.18% on Gram-positive bacterium *S. pyogenes* compared to 65.73% on Gram-negative bacterium *P. aeruginosa,* respectively, when treated with 100 μg/ml of ZnO NPs for 24 h (Liang et al., 2020; Dhanasegaran et al., 2022).

The structural and functional differences between the cell wall of Gram-positive and Gram-negative bacteria have been proposed as the major reason for the higher sensitivity of Gram-positive bacteria to ZnO NPs than Gram-negative bacteria (Aysa and Salman, 2016; Getie et al., 2017; Sukri et al., 2019; Demissie et al., 2020). The more complex cell wall of Gram-negative bacteria with the outer liposaccharide membrane makes the cell wall impermeable to antibacterial agents, whereas the peptidoglycan layer present in the Gram-positive bacteria is not an effective barrier against antibacterial agents, thus making them more susceptible to antimicrobials.

### Surface Interaction of ZnO NPs on Bacterial Cell Wall

**FIGURE 6:**
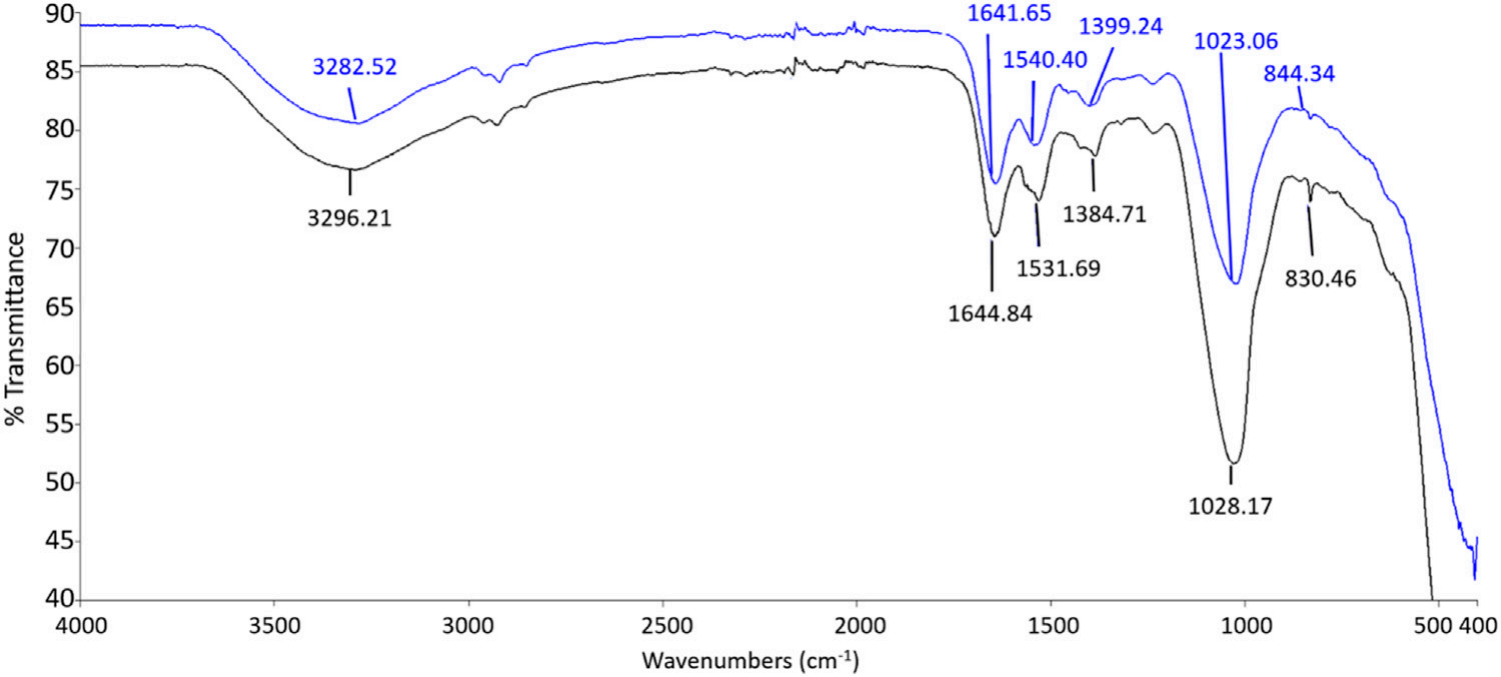
FTIR spectrum of *S. marcescens* as negative control (black line) and *S. marcescens* treated with 160 μg/ml of ZnO NPs for 24 h (blue line).

**FIGURE 7:**
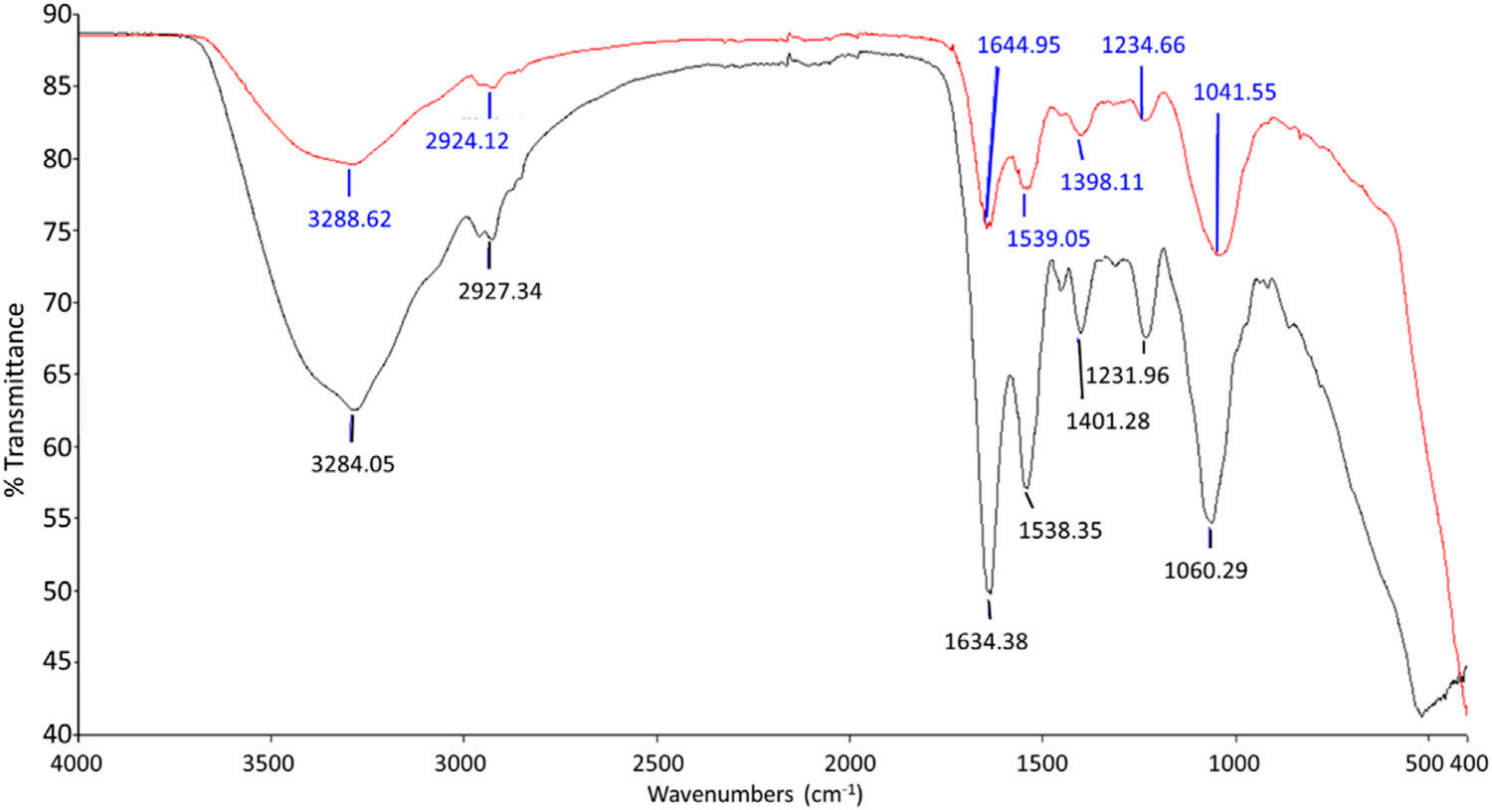
FTIR spectrum of negative control (black line) and *E. faecalis* treated with 160 μg/ml of ZnO NPs for 24 h (red line).

The FTIR spectrums displayed in Figure 6 and Figure 7 show the involvement of several functional groups for the interaction of ZnO NPs onto the cell wall of *S. marcescens* (Table 1) and *E. faecalis* (Table 2), respectively.

**TABLE 1:** The functional groups possibly involved in the binding of ZnO NPs onto the cell wall of *S. marcescens*.

Peak shift (Wave Number, cm^−1^)	Molecular motion	Functional group/biomolecule assignment
3296.21 3282.52	O―H stretching	Hydroxyl group
1644.84 1641.65	C=O stretching	Amide I
1531.69 1540.4	C―N stretching	Amide II
1384.71 1399.24	COO― stretching	Carboxyl group
1028.17 1023.06	C―O stretching	β 1,4 Glucans
830.46 844.3	α-Glycosidic linkage vibrations	Carbohydrates

**TABLE 2:** The functional groups possibly involved in the binding of ZnO NPs onto the cell wall of *E. faecalis*.

Peak shift (Wave number, cm^−1^)	Molecular motion	Functional group/biomolecule assignment
3284.05 3288.62	O-H stretching	Hydroxyl group
2927.34 2924.12	Stretching of CH_2_	Methylene group
1634.38 1644.95	C=O stretching	Amide I
1538.35 1539.05	N–H and C–N vibrations	Amide II
1401.25 1398.11	COO^−^ symmetric stretching	Carboxyl group
1231.96 1234.66	PO_2_^−^ asymmetric stretching	Phosphate
1060.29 1041.55	COH deformation	Mannans

The ZnO NPs treated *S. marcescens* showed the peak shifts from 3282.52 to 3296.21 cm^−1^, 1641.65 to 1644.84 cm^−1^, 1540.4 to 1531.69 cm^−1^, 1399.24 to 1384.71 cm^−1^, 1023.06 to 1028.17 cm^−1^, and 844.3 to 830.46 cm^−1^ corresponding to the stretching of O―H, C=O, C―N, COO―, C―O, and α-glycosidic linkage vibrations respectively.

A broad peak shift between 3282.52 and 3296.21 cm^−1^ indicated the O-H stretching due to the presence of the hydroxyl group (Nandiyanto et al., 2019). According to Ifeanyichukwu et al. (2020), the hydroxyl group on the bacterial cell surface can facilitate the binding of Zn ions to the bacterial surface. The peak shifts between 1641.65 to 1644.84 and 1531.69 to 1540.4 were associated with amide I and amide II from proteins, while the peak shift from 1384.71 to 1399.24 cm^−1^ corresponed to the carboxyl group of fatty acids and aminoacids (Ojeda et al., 2008; Liyanage et al., 2017; Nandiyanto et al., 2019). The peak shift beween 1023.06 and 1028.17 cm^−1^ exhibited the C-O stretching coupled with C-O bending of the C-OH of carbohydrates (Nandiyanto et al., 2019), especially β 1,4 glucans, and the shift from 844.3 to 830.46 cm^−1^ was possibly due to the α-glycosidic linkage vibrations of carbohydrates (Burattini et al., 2008).

Meanwhile, the peaks that shifted in ZnO NPs treated *E*. *faecalis* at 24 h were 3284.05–3288.62 cm^−1^ with O-H stretching, 2927.34 to 2924.12 cm^−1^ with CH_2_ stretching, 1634.38 to 1644.95 cm^−1^ with C=C stretching, 1231.96 to 1234.66 cm^−1^ with PO_2_^−^ asymmetric stretching, and 1060.29 to 1041.55 cm^−1^ due to COH deformation. The possible involvement of hydroxyl (3288.62 cm^−1^), methylene (2924.12 cm^−1^) group from lipids and proteins, amide I (1644.95 cm^−1^) from proteins, phosphate (1234.66 cm^−1^) from phospholipids and phosphated proteins, and mannans (1041.55 cm^−1^) (Burattini et al., 2008; Liyanage et al., 2017; Nandiyanto et al., 2019) of bacterial cell wall in the interaction of ZnO NPs onto the bacterial cell surface were determined.

Carboxylic groups on the cell wall contribute to the negative charge on the surfaces of bacteria cells. The strong electrostatic force at the negatively charged bacterial cells attracts the positively charged Zn ions to produce oxidative stress (Kim et al., 2020). Previous studies have acknowledged the existence of several functional groups such as carboxyl, hydroxyl, and amine groups on the bacterial cell surface which are predicted to bind with Zn ions (Yusof et al., 2020), and the involvement of proteins, polysaccharides, and lipids from the bacterial cell wall in the interaction of hematite-coated germanium crystal on bacteria (Ojeda et al., 2008). In this study, the hydroxyl, carboxyl, amides, methylene, and phosphate groups from the biomolecules such as proteins, carbohydrates, lipids, and phospholipids of bacterial cell wall are identified to be possibly involved in the interaction of ZnO NPs on the bacterial cell surface.

### Energy Dispersive X-Ray Analysis

**FIGURE 8:**
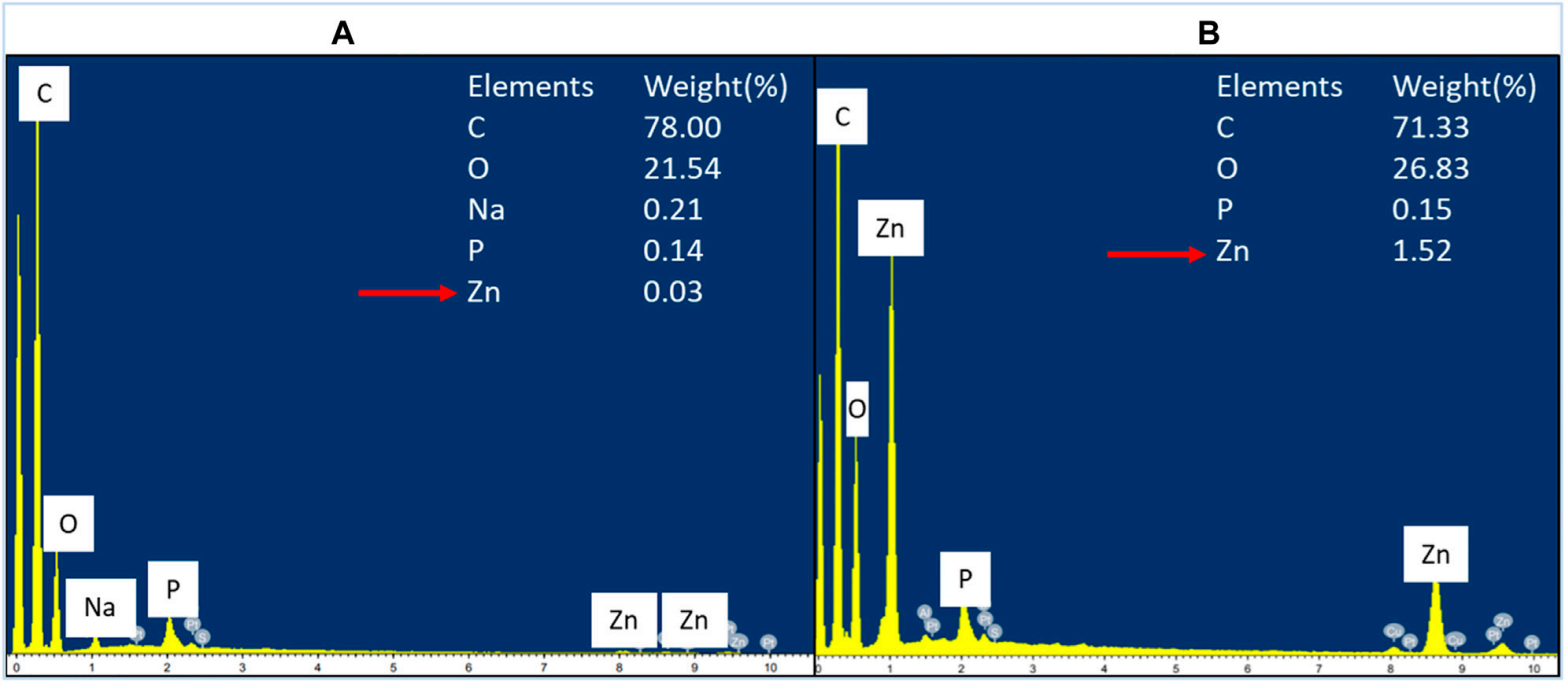
EDX spectrum of negative control of *E. faecalis* **(A)** and *E. faecalis* treated with 160 μg/ml of ZnO NPs for 24 h **(B)**.

**FIGURE 9:**
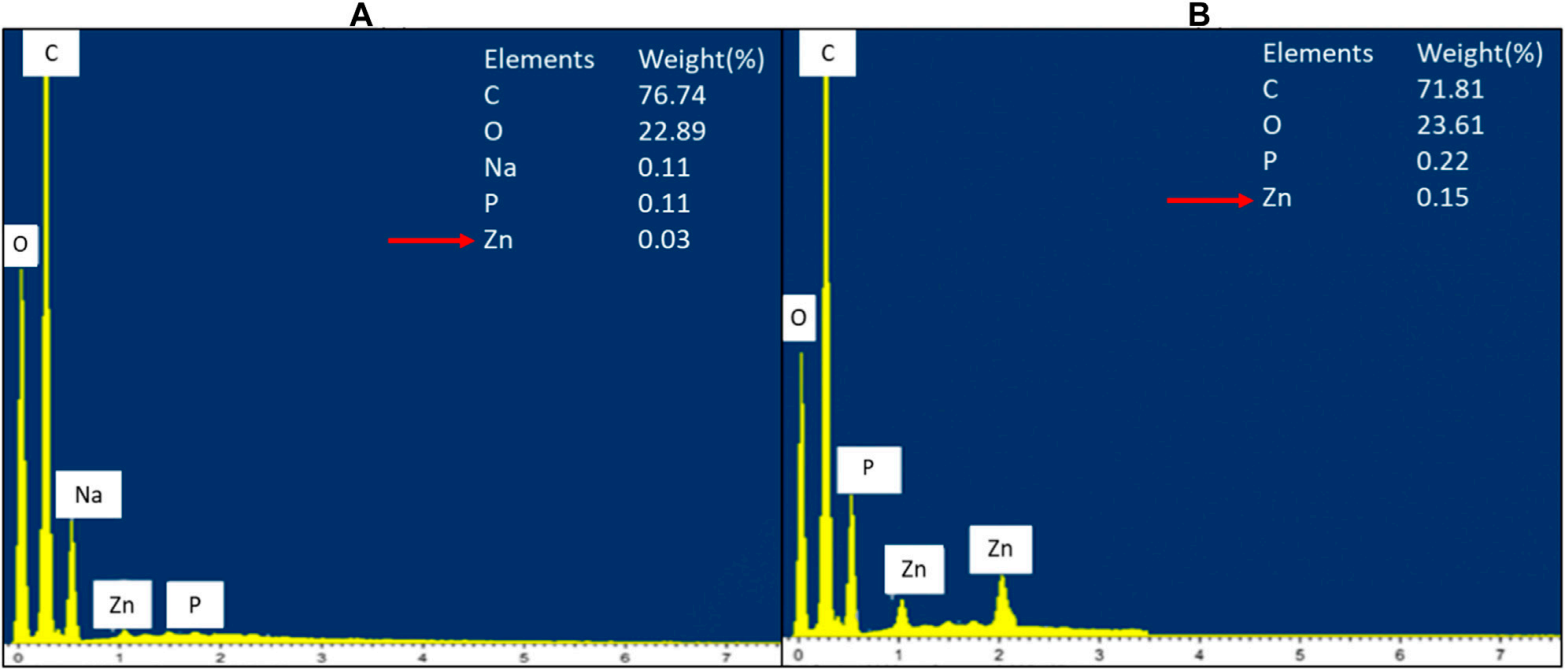
EDX spectrum of negative control of *S. marcescens* **(A)** and *S. marcescens* after treatment with 160 μg/ml of ZnO NPs for 24 h **(B)**.

The cellular accumulation of ZnO NPs on the treated bacteria was analyzed by EDX. The results demonstrated a higher accumulation of Zn in Gram-positive bacterium *E. faecalis* (Figure 8) compared to Gram-negative bacterium *S. marcescens* (Figure 9). The cellular accumulation of Zn corresponds to the bacterial growth inhibition reported in the present study. The higher percentage of growth inhibition reported on *E. faecalis* may be due to the higher accumulation of ZnO NPs in *E. faecalis*.

A study by Manda et al. (2016) reported a higher accumulation of silver NPs in *E. faecalis* compared to Gram-negative bacterium *P. vulgaris*. The less negative zeta potential charge on the cell wall of Gram-positive bacteria was reported to be responsible for the higher accumulation of silver NPs in Gram-positive bacteria compared to Gram-negative bacteria, which have more negative charge in the cell wall (Arakha et al., 2015; Manda et al. (2016). Similarly, ZnO NPs have negative zeta potential charge (Berg et al., 2009; El-Waseif, 2019), hence the occurence of less electrostatic repulsion between NPs and Gram-positive bacteria can cause higher uptake of NPs and subsequently higher growth inhibition in Gram-positive bacteria.

### Scanning Electron Microscopy

**FIGURE 10:**
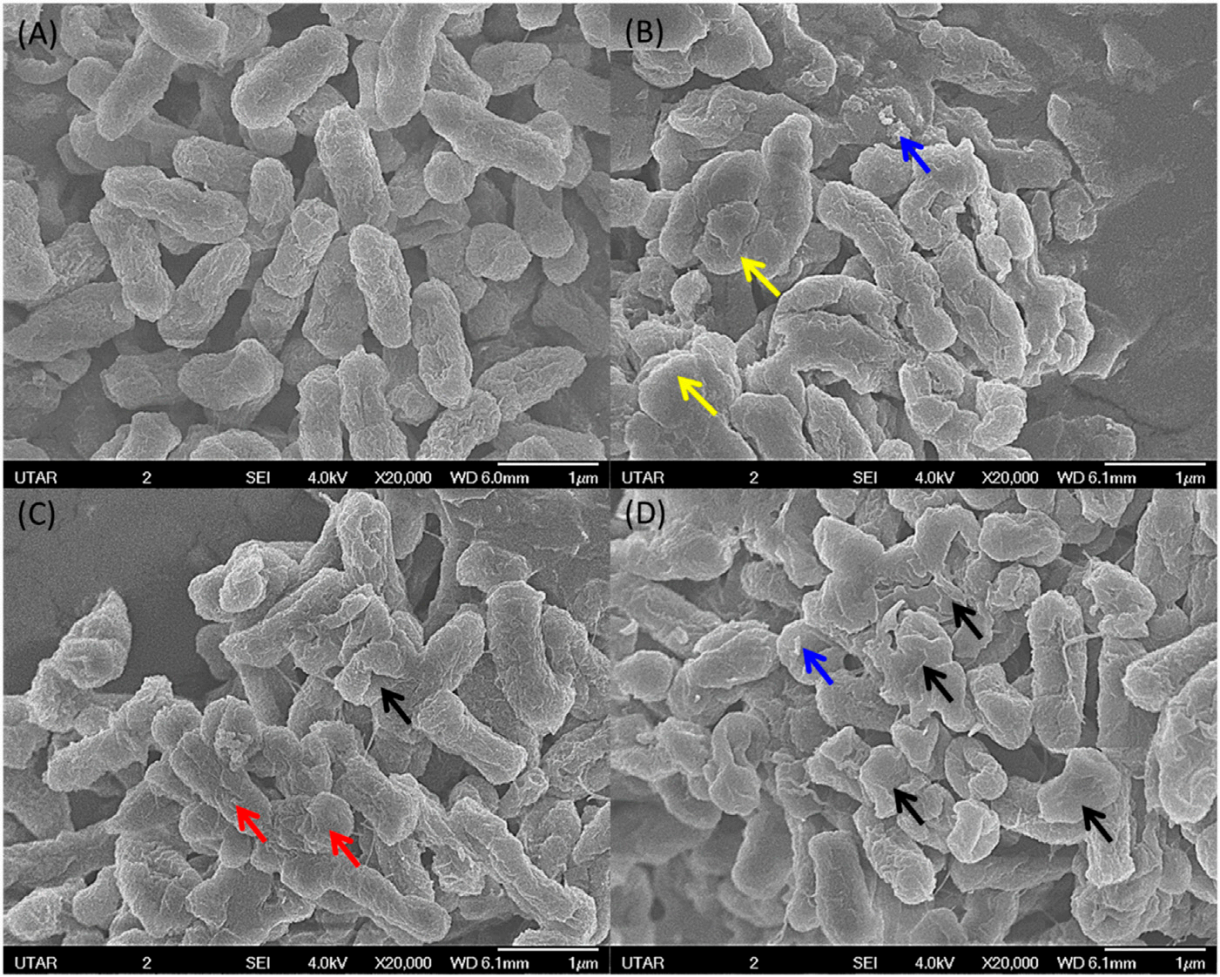
SEM image from negative control of *S. marcescens* with smooth surface and intact cell wall **(A)**. *S. marcescens* upon treatment with 160 μg/ml of ZnO NPs at 24 h **(B,C)** and **(D)** showing the attachment of NPs (blue arrow), wrinkled cell surface (red arrow), rupture of cell membrane (black arrow), and cell distortion (yellow arrow).

**FIGURE 11:**
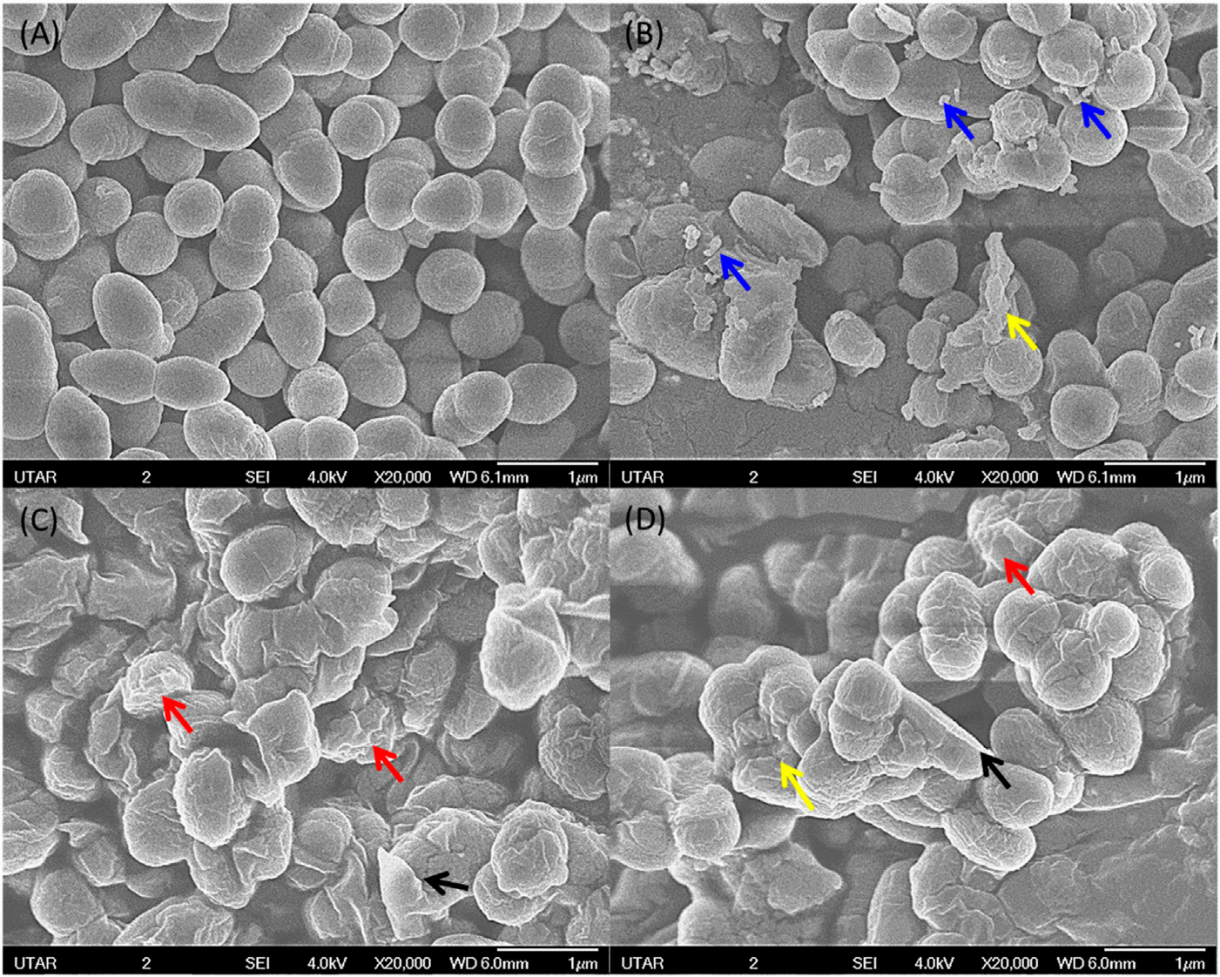
SEM image of negative control for *E. faecalis* with smooth surface and intact cell wall **(A)**. *E. faecalis* upon treatment with 160 μg/ml of ZnO NPs at 24 h **(B,C)** and **(D)** showing attachment of NPs (blue arrow), wrinkled cell surface (red arrow), rupture of cell membrane (black arrow), and cell distortion (yellow arrow).

The SEM image from the negative control of *S. marcescens* showed it as a rod-shaped smooth cell with intact cell wall. On the contrary, *S. marcescens* treated with ZnO NPs (160 μg/ml) for 24 h displayed the attachment of NPs on bacterial surface, rupture of cell membrane, and distortion of bacterial cells (Figure 10). Likewise, the negative control of *E. faecalis* showed smooth spherical cells with complete membrane, while *E. faecalis* treated with ZnO NPs showed attachment of NPs on bacteria, corrugations of cell membrane, and cell distortion (Figure 11).

The SEM images confirm the attachment of ZnO NPs on the bacterial cell surface and the subsequent disturbance to the integrity of bacterial cell membrane, which resulted in cell wrinkling and distortion. Similar findings were reported in the treatment of ZnO NPs, which caused cell membrane damage in *E. coli* (Arakha et al., 2015), cell shrinkage in *E. coli* and *S. enterica* (Bakur et al., 2019), membrane distortion, membrane blebbing, and leakage of cellular contents of *E. coli* and *P. mirabilis* (Maruthupandy et al., 2018).

The underlying reason that contributed to the cell rupture might be the production of reactive oxygen species due to the interaction of ZnO NPs on the bacterial cell surface, which can cause the loss of bacterial cell wall integrity by oxidizing the glutathione of bacterial cells to suppress their immune defense towards oxygen radicals (Siddiqi et al., 2018; Djearamane et al., 2020; Sánchez-López et al., 2020). According to Kairyte et al. (2013), treatment of ZnO NPs on *L. monocytogenes* resulted in bleb formation, which subsequently caused shrinkage of bacterial cells and intracellular component leakage, and eventually the death of bacterial cells. Further, our earlier studies have reported that the disruption in the cell wall integrity by the treatment of ZnO NPs caused cell death in *S. Pyogenes* and *P. aeruginosa,* respectively (Liang et al., 2020, and Dhanasekran et al., 2022).

**FIGURE 12:**
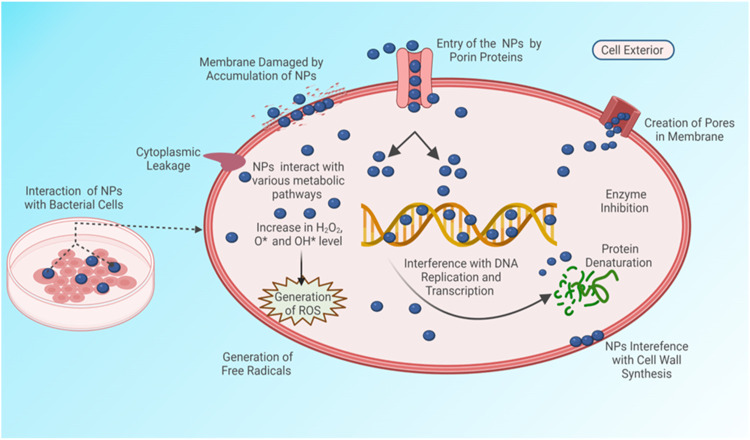
Antibacterial mechanisms of ZnO NPs.

Researchers have proposed various feasible mechanisms for the antibacterial properties of the metal and metal oxide NPs as illustrated in Figure 12 (Shaikh et al., 2019). Firstly, an increase in the production of reactive oxygen species by the interaction of NPs on bacteria can cause suppression of enzyme activity and ribosome disassembly, and lead to inactivation of protein synthesis, structural modification of essential proteins, and DNA damage, which eventually can result in the destruction of cellular components and cell death. Secondly, the disturbance in the cell membrane integrity due to the attachment of NPs on bacteria can increase the internalization of NPs and cause subsequent celluar damage (Shaikh et al., 2019; Lallo da Silva et al., 2019; Jiang et al., 2020). Finally, the dissolution of Zn ions from ZnO NPs can decrease the aminoacid metabolism and disturb the enzyme system, and cause loss in cell viability (Li et al., 2011; da Silva et al., 2019). Hence, the destruction of the bacteria cell wall observed from the present study might be due to the interaction of ZnO NPs on the bacterial surface and the subsequent induction of oxidative stress which resulted in the death of bacterial cells.

## Conclusion and Further Studies

The present study demonstrated a dose-dependent growth inhibitory effect of ZnO NPs on *S. marcescens* and *E. faecalis* at 24 h. The treatment of ZnO NPs caused a higher growth inhibition of 63.50 ± 2.50% on the Gram-positive bacterium *E*. *faecalis* compared with the Gram-negative bacterium *S. marcescens*, which had 51.27 ± 4.56% of growth reduction. The resuts also reported a higher accumulation of ZnO NPs on *E*. *faecalis* over *S. marcescens* which is believed to be the reason for the higher growth inhibition of *E. faecalis.* FTIR analysis identified the possible involvement of hydroxyl, carboxyl, amides, methylene, and phosphate groups from the biomolecules such as proteins, carbohydrates, lipids, and phospholipids of bacterial cell wall in the interaction of ZnO NPs on the bacterial surface. In addition, the morphological observations demonstrated that the exposure of *S. marcescens* and *E. faecalis* to ZnO NPs caused the rupture of cell membrane, corrugations, and distortion of cells that sequentially might have lead to cell death and growth inhibition. The present study findings propose the potential utilization of ZnO NPs as an antibacterial agent in treating infections caused by *S. marcescens* and *E. faecalis*. Further research is recommended to study the induction of oxidative stress in our test bacteria when exposed to ZnO NPs by investigating the level of reactive oxygen species and lipid peroxidation to correlate with the bacterial growth inhibition reported in this study. Further, the measurement of zeta potential is suggested to justify the occurence of less electrostatic repulsion between Gram-positive bacteria and ZnO NPs compared to Gram-negative bacteria.

## Data Availability

The raw data supporting the conclusions of this article will be made available by the authors, without undue reservation.

## Abbreviations

NPs, nanoparticles; Zn, zinc; ZnO NPs, zinc oxide nanoparticles; SEM, scanning electron microscope; EDX, energy dispersive X-ray; XRD, X-ray diffractometer; FTIR, Fourier transform infrared; LB, Luria-Bertani; PBS, phosphate buffer saline.

## References

[B1] Abreo E. Altier N. (2019). Pangenome of *Serratia marcescens* Strains from Nosocomial and Environmental Origins Reveals Different Populations and the Links between Them. Sci. Rep. 9 (1), 46–48. 10.1038/s41598-018-37118-0 30631083 PMC6328595

[B2] Alekish M. Ismail Z. B. Albiss B. Nawasrah S. (2018). *In Vitro* antibacterial Effects of Zinc Oxide Nanoparticles on Multiple Drug-Resistant Strains of *Staphylococcus aureus* and *Escherichia coli*: An Alternative Approach for Antibacterial Therapy of Mastitis in Sheep. Vet. World 11 (10), 1428–1432. 10.14202/vetworld.2018.1428-1432 30532497 PMC6247879

[B4] Arakha M. Saleem M. Mallick B. C. Jha S. (2015). The Effects of Interfacial Potential on Antimicrobial Propensity of ZnO Nanoparticle. Sci. Rep. 5 (1), 9578–9610. 10.1038/srep09578 25873247 PMC4397836

[B5] Aslam B. Wang W. Arshad M. I. Khurshid M. Muzammil S. Rasool M. H. (2018). Antibiotic Resistance: a Rundown of a Global Crisis. Infect. Drug Resist 11, 1645–1658. 10.2147/IDR.S173867 30349322 PMC6188119

[B6] Ayobami O. Willrich N. Reuss A. Eckmanns T. Markwart R. (2020). The Ongoing Challenge of Vancomycin-Resistant *Enterococcus Faecium* and *Enterococcus Faecalis* in Europe: an Epidemiological Analysis of Bloodstream Infections. Emerg. Microbes Infect. 9 (1), 1180–1193. 10.1080/22221751.2020.1769500 32498615 PMC7448851

[B7] Aysa-Lastra M. Salman H. D. (2016). Mexico, Families in. World Sci. News 33, 1–5. 10.1002/9781119085621.wbefs132

[B8] Azam A. Ahmed A. S. Oves M. Khan M. S. Habib S. S. Memic A. (2012). Antimicrobial Activity of Metal Oxide Nanoparticles against Gram-Positive and Gram-Negative Bacteria: a Comparative Study. Int. J. Nanomedicine 7, 6003–6009. 10.2147/IJN.S35347 23233805 PMC3519005

[B9] Bakur A. Elshaarani T. Niu Y. Chen Q. (2019). Comparative Study of Antidiabetic, Bactericidal, and Antitumor Activities of MEL@AgNPs, MEL@ZnONPs, and Ag-ZnO/MEL/GA Nanocomposites Prepared by Using MEL and Gum Arabic. RSC Adv. 9 (17), 9745–9754. 10.1039/c9ra00344d 35520700 PMC9062402

[B10] Burattini E. Cavagna M. Dell’Anna R. Malvezzi Campeggi F. Monti F. Rossi F. (2008). A FTIR Microspectroscopy Study of Autolysis in Cells of the Wine Yeast Saccharomyces cerevisiae. Vib. Spectrosc. 47 (2), 139–147. 10.1016/j.vibspec.2008.04.007

[B11] Castilho A. L. Saraceni C. H. Díaz I. E. Paciencia M. L. Suffredini I. B. (2013). New Trends in Dentistry: Plant Extracts against *Enterococcus faecalis*. The Efficacy Compared to Chlorhexidine. Braz Oral Res. 27 (2), 109–115. 10.1590/s1806-83242013000100017 23538423

[B12] Chandra H. Patel D. Kumari P. Jangwan J. S. Yadav S. (2019). Phyto-mediated Synthesis of Zinc Oxide Nanoparticles of *Berberis Aristata*: Characterization, Antioxidant Activity and Antibacterial Activity with Special Reference to Urinary Tract Pathogens. Mater Sci. Eng. C Mater Biol. Appl. 102, 212–220. 10.1016/j.msec.2019.04.035 31146992

[B13] Conceição N. da Silva L. E. Darini A. L. Pitondo-Silva A. de Oliveira A. G. (2014). Penicillin-resistant, Ampicillin-Susceptible *Enterococcus faecalis* of Hospital Origin: Pbp4 Gene Polymorphism and Genetic Diversity. Infect. Genet. Evol. 28, 289–295. 10.1016/j.meegid.2014.10.018 25445645

[B14] Dadi R. Azouani R. Traore M. Mielcarek C. Kanaev A. (2019). Antibacterial Activity of ZnO and CuO Nanoparticles against Gram Positive and Gram Negative Strains. Mater Sci. Eng. C Mater Biol. Appl. 104, 109968–109969. 10.1016/j.msec.2019.109968 31500003

[B15] Demissie M. G. Sabir F. K. Edossa G. D. Gonfa B. A. (2020). Synthesis of Zinc Oxide Nanoparticles Using Leaf Extract of *Lippia Adoensis* (Koseret) and Evaluation of its Antibacterial Activity. J. Chem. 2020, 7459042. 10.1155/2020/7459042

[B16] Dhanasegaran K. Djearamane S. Liang S. X. T. Wong L. S. Kasivelu G. Lee P. F. (2022). Antibacterial Properties of Zinc Oxide Nanoparticles on *Pseudomonas aeruginosa* (ATCC 27853). Sci. Iran. 28 (6), 3806. 10.24200/sci.2021.56815.4974

[B17] Dhusia K. Raja K. Thomas P. P. M. Yadav P. K. Ramteke P. W. (2019). Molecular Dynamics Simulation Analysis of Conessine against Multi Drug Resistant *Serratia marcescens* . Infect. Genet. Evol. 67, 101–111. 10.1016/j.meegid.2018.11.001 30396000

[B18] Dikshit P. Kumar J. Das A. Sadhu S. Sharma S. Singh S. (2021). Green Synthesis of Metallic Nanoparticles: Applications and Limitations. Catalysts 11 (902), 902–937. 10.3390/catal11080902

[B19] Divya M. Vaseeharan B. Abinaya M. Vijayakumar S. Govindarajan M. Alharbi N. S. (2018). Biopolymer Gelatin-Coated Zinc Oxide Nanoparticles Showed High Antibacterial, Antibiofilm and Anti-angiogenic Activity. J. Photochem Photobiol. B 178, 211–218. 10.1016/j.jphotobiol.2017.11.008 29156349

[B20] Djearamane S. Wong L. S. Lim Y. M. Lee P. F. (2019). Cytotoxic Effects of Zinc Oxide Nanoparticles on *Chlorella Vulgaris* . Poll. Res. 38 (2), 479–484. http://www.envirobiotechjournals.com/article_abstract.php?aid=9594&iid=274&jid=4.

[B21] Djearamane S. Wong L. S. Lim Y. M. Lee P. F. (2020). Oxidative Stress Effects of Zinc Oxide Nanoparticles on Fresh Water Microalga *Haematococcus pluvialis* . Ecol. Environ. Conservation 26 (2), 663

[B22] Dowlatababdi F. H. Amiri G. Sichani M. M. (2017). Investigation of the Antimicrobial Effect of Silver Doped Zinc Oxide Nanoparticles. Nanomed J. 4 (1), 50–54. 10.22038/NMJ.2017.8053

[B23] Fair R. J. Tor Y. (2014). Antibiotics and Bacterial Resistance in the 21st Century. Perspect. Med. Chem. 6, 25–64. 10.4137/PMC.S14459 PMC4159373 25232278

[B24] Fernando S. Gunasekara T. Holton J. (2018). Antimicrobial Nanoparticles: Applications and Mechanisms of Action. Sri Lankan J. Infec Dis. 8 (1), 2. 10.4038/sljid.v8i1.8167

[B25] Fisher M. C. Gurr S. J. Cuomo C. A. Blehert D. S. Jin H. Stukenbrock E. H. (2020). Threats Posed by the Fungal Kingdom to Humans, Wildlife, and Agriculture. mBio 11 (3), e00449–20. 10.1128/mBio.00449-20 32371596 PMC7403777

[B26] Getie S. Belay A. Chandra Reddy A. R. Belay Z. (2017). Synthesis and Characterizations of Zinc Oxide Nanoparticles for Antibacterial Applications. J. Nanomed Nanotechno S 8 (004), s8. 10.4172/2157-7439.S8-004

[B27] Gunalan S. Sivaraj R. Rajendran V. (2012). Green Synthesized ZnO Nanoparticles against Bacterial and Fungal Pathogens. Prog. Nat. Sci. Mater. Int. 22 (6), 693–700. 10.1016/j.pnsc.2012.11.015

[B28] Ifeanyichukwu U. L. Fayemi O. E. Ateba C. N. (2020). Green Synthesis of Zinc Oxide Nanoparticles from Pomegranate (*Punica Granatum*) Extracts and Characterization of Their Antibacterial Activity. Molecules 25 (19), 1–22. 10.3390/molecules25194521 PMC7583900 33023149

[B29] Jabbari Shiadeh S. M. Pormohammad A. Hashemi A. Lak P. (2019). Global Prevalence of Antibiotic Resistance in Blood-Isolated *Enterococcus faecalis* and *Enterococcus Faecium*: a Systematic Review and Meta-Analysis. Infect. Drug Resist 12, 2713–2725. 10.2147/IDR.S206084 31564921 PMC6731464

[B65] Jiang S. Lin K. Cai M. (2020). ZnO Nanomaterials: Current Advancements in Antibacterial Mechanisms and Applications. Front. Chem. 8, 580. 10.3389/fchem.2020.00580 32793554 PMC7385224

[B30] Kadiyala U. Turali-Emre E. S. Bahng J. H. Kotov N. A. VanEpps J. S. (2018). Unexpected Insights into Antibacterial Activity of Zinc Oxide Nanoparticles against Methicillin Resistant *Staphylococcus aureus* (MRSA). Nanoscale 10, 4927–4939. 10.1039/C7NR08499D 29480295 PMC5847298

[B31] Kairyte K. Kadys A. Luksiene Z. (2013). Antibacterial and Antifungal Activity of Photoactivated ZnO Nanoparticles in Suspension. J. Photochem Photobiol. B 128, 78–84. 10.1016/j.jphotobiol.2013.07.017 24035847

[B32] Kau A. L. Martin S. M. Lyon W. Hayes E. Caparon M. G. Hultgren S. J. (2005). *Enterococcus faecalis* Tropism for the Kidneys in the Urinary Tract of C57BL/6J Mice. Infect. Immun. 73 (4), 2461–2468. 10.1128/IAI.73.4.2461-2468.2005 15784592 PMC1087416

[B33] Khayyat A. N. Hegazy W. A. H. Shaldam M. A. Mosbah R. Almalki A. J. Ibrahim T. S. Khayat M. T. Khafagy E. S. Soliman W. E. Abbas H. A. (2021). Xylitol Inhibits Growth and Blocks Virulence in *Serratia marcescens* . Microorganisms 9 (5), 1083. 10.3390/microorganisms9051083 34070043 PMC8158113

[B34] Kim I. Viswanathan K. Kasi G. Sadeghi K. Thanakkasaranee S. Seo J. (2020). Preparation and Characterization of Positively Surface Charged Zinc Oxide Nanoparticles against Bacterial Pathogens. Microb. Pathog. 149, 104290–104299. 10.1016/j.micpath.2020.104290 32492458

[B35] Kim S. B. Jeon Y. D. Kim J. H. Kim J. K. Ann H. W. Choi H. (2015). Risk Factors for Mortality in Patients with *Serratia marcescens* Bacteremia. Yonsei Med. J. 56 (2), 348–354. 10.3349/ymj.2015.56.2.348 25683980 PMC4329343

[B36] Kumar H. Rani R. (2013). Structural and Optical Characterization of ZnO Nanoparticles Synthesized by Microemulsion Route. Int. Lett. Chem. Phys. Astronomy 14, 26. 10.18052/www.scipress.com/ilcpa.19.26

[B37] Kumar P. Gautam S. (2019). *Developing ZnO Nanoparticle Embedded* Antimicrobial Starch Biofilm for Food Packaging. *Cornell University [preprint]* . Available at: https://arxiv.org/abs/1909.05083 (Accessed 01 May, 2022).

[B38] Lallo da Silva B. Abuçafy M. P. Berbel Manaia E. Oshiro Junior J. A. Chiari-Andréo B. G. Pietro R. C. R. (2019). Relationship between Structure and Antimicrobial Activity of Zinc Oxide Nanoparticles: An Overview. Int. J. Nanomedicine 14, 9395–9410. 10.2147/IJN.S216204 31819439 PMC6897062

[B39] Lallo da Silva B. Caetano B. L. Chiari-Andréo B. G. Pietro R. C. L. R. Chiavacci L. A. (2019). Increased Antibacterial Activity of ZnO Nanoparticles: Influence of Size and Surface Modification. Colloids Surf. B Biointerfaces 177, 440–447. 10.1016/j.colsurfb.2019.02.013 30798065

[B40] Li M. Zhu L. Lin D. (2011). Toxicity of ZnO Nanoparticles to *Escherichia coli*: Mechanism and the Influence of Medium Components. Environ. Sci. Technol. 45 (5), 1977–1983. 10.1021/es102624t 21280647

[B41] Liang S. X. T. Wong L. S. Lim Y. M. Lee P. F. Djearamane S. (2020). Effects of Zinc Oxide Nanoparticles on *Streptococcus Pyogenes* . South Afr. J. Chem. Eng. 34, 63–71. 10.1016/j.sajce.2020.05.009

[B42] Liyanage S. Dassanayake R. S. Bouyanfif A. Rajakaruna E. Ramalingam L. Moustaid-Moussa N. (2017). Optimization and Validation of Cryostat Temperature Conditions for Trans-reflectance Mode FTIR Microspectroscopic Imaging of Biological Tissues. MethodsX 4, 118–127. 10.1016/j.mex.2017.01.006 28280690 PMC5333507

[B43] Maheswaran H. Wong L. S. Narendhirakannan R. T. Janakiraman A. K. Djearamane S. (2021). Toxicity of Zinc Oxide Nanoparticles on Human Skin Dermal Cells. J. Exp. Biol. Agric. Sci. 9 (Spl-1-GCSGD_2020), S95–S100. 10.18006/2021.9(spl-1-gcsgd_2020).s95.s100

[B44] Maruthupandy M. Rajivgandhi G. Muneeswaran T. Song J. M. Manoharan N. (2018). Biologically Synthesized Zinc Oxide Nanoparticles as Nanoantibiotics against ESBLs Producing Gram Negative Bacteria. Microb. Pathog. 121, 224–231. 10.1016/j.micpath.2018.05.041 29807135

[B45] Mohd Yusof H. Mohamad R. Zaidan U. H. Rahman N. A. (2020). Sustainable Microbial Cell Nanofactory for Zinc Oxide Nanoparticles Production by Zinc-Tolerant Probiotic *Lactobacillus Plantarum* Strain TA4. Microb. Cell Fact. 19 (1), 10–17. 10.1186/s12934-020-1279-6 31941498 PMC6964013

[B46] Muhammad W. Ullah N. Haroon M. Abbasi B. H. (2019). Optical, Morphological and Biological Analysis of Zinc Oxide Nanoparticles (ZnO NPs) Using *Papaver Somniferum* L. RSC Adv. 9 (51), 29541–29548. 10.1039/c9ra04424h 35531532 PMC9071912

[B47] Nandiyanto A. B. D. Oktiani R. Ragadhita R. (2019). How to Read and Interpret FTIR Spectroscope of Organic Material. Indones. J. Sci. Technol. 4 (1), 97–118. 10.17509/ijost.v4i1.15806

[B48] Nilavukkarasi M. Vijayakumar S. Prathipkumar S. (2020). Capparis Zeylanica Mediated Bio-Synthesized ZnO Nanoparticles as Antimicrobial, Photocatalytic and Anti-cancer Applications. Mater. Sci. Energy Technol. 3, 335–343. 10.1016/j.mset.2019.12.004

[B49] Ojeda J. J. Romero-Gonzalez M. E. Pouran H. M. Banwart S. A. (2008). *In Situ* monitoring of the Biofilm Formation of *Pseudomonas Putida* on Hematite Using Flow-Cell ATR-FTIR Spectroscopy to Investigate the Formation of Inner-Sphere Bonds between the Bacteria and the Mineral. Mineral. Mag. 72 (1), 101–106. 10.1180/minmag.2008.072.1.101

[B50] Premanathan M. Karthikeyan K. Jeyasubramanian K. Manivannan G. (2012). Selective Toxicity of ZnO Nanoparticles toward Gram-Positive Bacteria and Cancer Cells by Apoptosis through Lipid Peroxidation. Nanomedicine 7, 184–192. 10.1016/j.nano.2010.10.001 21034861

[B51] Ramesh M. Anbuvannan M. ViruthagirI G. (2015). Green Synthesis of ZnO Nanoparticles Using *Solanum nigrum* Leaf Extract and Their Antibacterial Activity. Spectrochim. Acta A Mol. Biomol. Spectrosc. 136, 864–870. 10.1016/j.saa.2014.09.105 25459609

[B52] Ray C. Shenoy A. T. Orihuela C. J. González-Juarbe N. (2017). Killing of *Serratia marcescens* Biofilms with Chloramphenicol. Ann. Clin. Microbiol. Antimicrob. 16 (1), 19. 10.1186/s12941-017-0192-2 28356113 PMC5370475

[B53] Ray U. Dutta S. Chakravarty C. Sutradhar A. (2015). A Case of Multiple Cutaneous Lesions Due to *Serratia marcescens* in an Immunocompromised Patient. JMM Case Rep. 2 (3), e000059. 10.1099/jmmcr.0.000059

[B54] Sánchez-López E. Gomes D. Esteruelas G. Bonilla L. Lopez-Machado A. L. Galindo R. (2020). Metal-Based Nanoparticles as Antimicrobial Agents: An Overview. Nanomater. (Basel) 10 (292), 1–39. 10.3390/nano10020292 PMC7075170 32050443

[B55] Seil J. T. Webster T. J. (2012). Antimicrobial Applications of Nanotechnology: Methods and Literature. Int. J. Nanomedicine 7, 2767–2781. 10.2147/IJN.S24805 22745541 PMC3383293

[B66] Shaikh S. Nazam N. Rizvi S.M.D. Ahmad K. Baig M.H. Lee E.J. Choi I. (2019). Mechanistic insights into the antimicrobial actions of metallic nanoparticles and their implications for multidrug resistance. Int. J. Mol. Sci. 20 (10), 2468. 10.3390/ijms20102468 PMC6566786 31109079

[B56] Siddiqi K. S. Rahman A. Husen A. (2018). Properties of Zinc Oxide Nanoparticles and Their Activity against Microbes. Nanoscale Res. Lett. 13 (1), 1–13. 10.1186/s11671-018-2532-3 29740719 PMC5940970

[B57] Sirelkhatim A. Mahmud S. Seeni A. Kaus N. H. M. Ann L. C. Bakhori S. K. M. (2015). Review on Zinc Oxide Nanoparticles: Antibacterial Activity and Toxicity Mechanism. Nanomicro Lett. 7, 219–242. 10.1007/s40820-015-0040-x 30464967 PMC6223899

[B58] Sukri S. N. A. M. Shameli K. Wong M. M. T. Teow S. Y. Chew J. Ismail N. A. (2019). Cytotoxicity and Antibacterial Activities of Plant-Mediated Synthesized Zinc Oxide (ZnO) Nanoparticles Using *Punica Granatum* (Pomegranate) Fruit Peels Extract. J. Mol. Struct. 1189, 57. 10.1016/j.molstruc.2019.04.026

[B59] Tiwari V. Mishra N. Gadani K. Solanki P. S. Shah N. A. Tiwari M. (2018). Mechanism of Anti-bacterial Activity of Zinc Oxide Nanoparticle against Carbapenem-Resistant *Acinetobacter Baumannii* . Front. Microbiol. 9, 1218. 10.3389/fmicb.2018.01218 29928271 PMC5997932

[B60] Varadavenkatesan T. Lyubchik E. Pai S. Pugazhendhi A. Vinayagam R. Selvaraj R. (2019). Photocatalytic Degradation of Rhodamine B by Zinc Oxide Nanoparticles Synthesized Using the Leaf Extract of *Cyanometra Ramiflora* . J. Photochem Photobiol. B 199, 111621–111628. 10.1016/j.jphotobiol.2019.111621 31610434

[B61] Ventola C. L. (2015). The Antibiotic Resistance Crisis: Part 1: Causes and Threats. P T 40 (4), 277. https://www.ncbi.nlm.nih.gov/pmc/articles/PMC4378521/. 25859123 PMC4378521

[B62] Vijayakumar S. Krishnakumar C. Arulmozhi P. Mahadevan S. Parameswari N. (2018). Biosynthesis, Characterization and Antimicrobial Activities of Zinc Oxide Nanoparticles from Leaf Extract of *Glycosmis Pentaphylla* (Retz.) DC. Microb. Pathog. 116, 44–48. 10.1016/j.micpath.2018.01.003 29330059

[B63] Wang L. Hu C. Shao L. (2017). The Antimicrobial Activity of Nanoparticles: Present Situation and Prospects for the Future. Int. J. Nanomedicine 12, 1227–1249. 10.2147/IJN.S121956 28243086 PMC5317269

[B64] Yaqoob A. A. Ahmad H. Parveen T. Ahmad A. Oves M. Ismail I. M. I. (2020). Recent Advances in Metal Decorated Nanomaterials and Their Various Biological Applications: A Review. Front. Chem. 8, 341–423. 10.3389/fchem.2020.00341 32509720 PMC7248377

